# A systematic review to determine the effect of strategies to sustain chronic disease prevention interventions in clinical and community settings

**DOI:** 10.1093/tbm/ibae070

**Published:** 2025-01-22

**Authors:** Edward Riley-Gibson, Alix Hall, Adam Shoesmith, Luke Wolfenden, Rachel C Shelton, William Pascoe, Belinda Peden, Emma Doherty, Emma Pollock, Debbie Booth, Ramzi G Salloum, Celia Laur, Byron J Powell, Melanie Kingsland, Cassandra Lane, Maji Hailemariam, Rachel Sutherland, Nicole Nathan

**Affiliations:** School of Medicine and Public Health, The University of Newcastle, Newcastle, University Drive, Callaghan, 2308 New South Wales, Australia; The National Centre of Implementation Science (NCOIS), The University of Newcastle, University Drive, Callaghan, 2308 Newcastle, New South Wales, Australia; Hunter Medical Research Institute, HMRI Building, Lot 1, Kookaburra Circuit, New Lambton Heights, 2305 New South Wales, Australia; Hunter New England Population Health, Hunter New England Local Health District, Locked Bag 10, Wallsend, 2287 Newcastle, New South Wales, Australia; School of Medicine and Public Health, The University of Newcastle, Newcastle, University Drive, Callaghan, 2308 New South Wales, Australia; The National Centre of Implementation Science (NCOIS), The University of Newcastle, University Drive, Callaghan, 2308 Newcastle, New South Wales, Australia; Hunter Medical Research Institute, HMRI Building, Lot 1, Kookaburra Circuit, New Lambton Heights, 2305 New South Wales, Australia; Hunter New England Population Health, Hunter New England Local Health District, Locked Bag 10, Wallsend, 2287 Newcastle, New South Wales, Australia; School of Medicine and Public Health, The University of Newcastle, Newcastle, University Drive, Callaghan, 2308 New South Wales, Australia; The National Centre of Implementation Science (NCOIS), The University of Newcastle, University Drive, Callaghan, 2308 Newcastle, New South Wales, Australia; Hunter Medical Research Institute, HMRI Building, Lot 1, Kookaburra Circuit, New Lambton Heights, 2305 New South Wales, Australia; Hunter New England Population Health, Hunter New England Local Health District, Locked Bag 10, Wallsend, 2287 Newcastle, New South Wales, Australia; School of Medicine and Public Health, The University of Newcastle, Newcastle, University Drive, Callaghan, 2308 New South Wales, Australia; The National Centre of Implementation Science (NCOIS), The University of Newcastle, University Drive, Callaghan, 2308 Newcastle, New South Wales, Australia; Hunter Medical Research Institute, HMRI Building, Lot 1, Kookaburra Circuit, New Lambton Heights, 2305 New South Wales, Australia; Hunter New England Population Health, Hunter New England Local Health District, Locked Bag 10, Wallsend, 2287 Newcastle, New South Wales, Australia; Department of Sociomedical Sciences, Mailman School of Public Health, Columbia University, 722 West 168th Street, New York, NY 10032, USA; School of Medicine and Public Health, The University of Newcastle, Newcastle, University Drive, Callaghan, 2308 New South Wales, Australia; The National Centre of Implementation Science (NCOIS), The University of Newcastle, University Drive, Callaghan, 2308 Newcastle, New South Wales, Australia; Hunter Medical Research Institute, HMRI Building, Lot 1, Kookaburra Circuit, New Lambton Heights, 2305 New South Wales, Australia; Hunter New England Population Health, Hunter New England Local Health District, Locked Bag 10, Wallsend, 2287 Newcastle, New South Wales, Australia; School of Medicine and Public Health, The University of Newcastle, Newcastle, University Drive, Callaghan, 2308 New South Wales, Australia; The National Centre of Implementation Science (NCOIS), The University of Newcastle, University Drive, Callaghan, 2308 Newcastle, New South Wales, Australia; Hunter Medical Research Institute, HMRI Building, Lot 1, Kookaburra Circuit, New Lambton Heights, 2305 New South Wales, Australia; Hunter New England Population Health, Hunter New England Local Health District, Locked Bag 10, Wallsend, 2287 Newcastle, New South Wales, Australia; School of Medicine and Public Health, The University of Newcastle, Newcastle, University Drive, Callaghan, 2308 New South Wales, Australia; The National Centre of Implementation Science (NCOIS), The University of Newcastle, University Drive, Callaghan, 2308 Newcastle, New South Wales, Australia; Hunter Medical Research Institute, HMRI Building, Lot 1, Kookaburra Circuit, New Lambton Heights, 2305 New South Wales, Australia; Hunter New England Population Health, Hunter New England Local Health District, Locked Bag 10, Wallsend, 2287 Newcastle, New South Wales, Australia; School of Medicine and Public Health, The University of Newcastle, Newcastle, University Drive, Callaghan, 2308 New South Wales, Australia; The National Centre of Implementation Science (NCOIS), The University of Newcastle, University Drive, Callaghan, 2308 Newcastle, New South Wales, Australia; Hunter Medical Research Institute, HMRI Building, Lot 1, Kookaburra Circuit, New Lambton Heights, 2305 New South Wales, Australia; Hunter New England Population Health, Hunter New England Local Health District, Locked Bag 10, Wallsend, 2287 Newcastle, New South Wales, Australia; School of Medicine and Public Health, The University of Newcastle, Newcastle, University Drive, Callaghan, 2308 New South Wales, Australia; The National Centre of Implementation Science (NCOIS), The University of Newcastle, University Drive, Callaghan, 2308 Newcastle, New South Wales, Australia; Hunter Medical Research Institute, HMRI Building, Lot 1, Kookaburra Circuit, New Lambton Heights, 2305 New South Wales, Australia; Hunter New England Population Health, Hunter New England Local Health District, Locked Bag 10, Wallsend, 2287 Newcastle, New South Wales, Australia; Department of Health Outcomes & Biomedical Informatics, University of Florida College of Medicine, PO Box 100177, Gainesville, FL 32610-0177, USA; Women’s College Hospital Institute for Health System Solutions and Virtual Care, 76 Grenville Street, Toronto, Ontario M5S 1B2, Canada; Institute of Health Policy, Management and Evaluation, University of Toronto, Health Sciences Building, 155 College Street, Suite 425, Toronto, Ontario M5T 3M6, Canada; Center for Mental Health Services Research, Brown School, Washington University in St. Louis, 600 S. Taylor Avenue, Suite 100, St. Louis, MO 63110, USA; Center for Dissemination and Implementation, Institute for Public Health, Washington University in St. Louis, 600 S. Taylor Avenue, Suite 100, St. Louis, MO 63110, USA; Division of Infectious Diseases, John T. Milliken Department of Medicine, School of Medicine, Washington University in St. Louis, 660 S. Euclid Avenue, Campus Box 8051, St. Louis, MO 63110, USA; School of Medicine and Public Health, The University of Newcastle, Newcastle, University Drive, Callaghan, 2308 New South Wales, Australia; Hunter Medical Research Institute, HMRI Building, Lot 1, Kookaburra Circuit, New Lambton Heights, 2305 New South Wales, Australia; Hunter New England Population Health, Hunter New England Local Health District, Locked Bag 10, Wallsend, 2287 Newcastle, New South Wales, Australia; School of Medicine and Public Health, The University of Newcastle, Newcastle, University Drive, Callaghan, 2308 New South Wales, Australia; The National Centre of Implementation Science (NCOIS), The University of Newcastle, University Drive, Callaghan, 2308 Newcastle, New South Wales, Australia; Hunter Medical Research Institute, HMRI Building, Lot 1, Kookaburra Circuit, New Lambton Heights, 2305 New South Wales, Australia; Hunter New England Population Health, Hunter New England Local Health District, Locked Bag 10, Wallsend, 2287 Newcastle, New South Wales, Australia; Charles Stewart Mott Department of Public Health, College of Human Medicine, Michigan State University, 200 E. First Street, Flint, MI 48502, USA; School of Medicine and Public Health, The University of Newcastle, Newcastle, University Drive, Callaghan, 2308 New South Wales, Australia; The National Centre of Implementation Science (NCOIS), The University of Newcastle, University Drive, Callaghan, 2308 Newcastle, New South Wales, Australia; Hunter Medical Research Institute, HMRI Building, Lot 1, Kookaburra Circuit, New Lambton Heights, 2305 New South Wales, Australia; Hunter New England Population Health, Hunter New England Local Health District, Locked Bag 10, Wallsend, 2287 Newcastle, New South Wales, Australia; School of Medicine and Public Health, The University of Newcastle, Newcastle, University Drive, Callaghan, 2308 New South Wales, Australia; The National Centre of Implementation Science (NCOIS), The University of Newcastle, University Drive, Callaghan, 2308 Newcastle, New South Wales, Australia; Hunter Medical Research Institute, HMRI Building, Lot 1, Kookaburra Circuit, New Lambton Heights, 2305 New South Wales, Australia; Hunter New England Population Health, Hunter New England Local Health District, Locked Bag 10, Wallsend, 2287 Newcastle, New South Wales, Australia

**Keywords:** implementation science, sustainment, sustainability, maintenance, implementation, evidence-based intervention, chronic disease prevention, implementation strategies, sustainment strategies

## Abstract

This review assessed the effect of strategies designed to sustain the delivery of evidenced based interventions (EBIs) which target behavioural risk factors linked to leading causes of chronic disease in clinical and community settings. Seven electronic databases were searched for randomised controlled studies published from earliest record to November 2022. Studies were included if they tested a strategy to sustain the delivery of an EBI within clinical or community settings. Results were synthesised using vote counting based on direction of effect, and reported in accordance with non-meta-analytic review standards following the Synthesis Without Meta-analysis (SWiM) guidelines. Three studies met the study inclusion criteria. Two studies were community-based, with one conducted in Australian community sports clubs and the second in afterschool clubs in the United States. The single clinical-based study was conducted in community health care centres in the United States. Across the three studies, 25 strategies were employed and only two strategies were common across all studies. Synthesis using vote counting based on direction of effect indicated that two of three studies favoured the intervention as positively impacting sustainment of EBIs. Few studies have been conducted to assess the effect of strategies designed to support sustainment of EBIs for chronic disease prevention in clinical and community settings. As such, it is difficult to determine the effect of strategies designed to support sustainment. Further research with comprehensive reporting of the selection, use and testing of sustainment strategies is needed to advance understanding of how to sustain EBIs in clinical and community settings.

## Teaser

Strategies that target a wide range of barriers may be most effective in supporting the sustainment of health programmes.

**Implications**

**Practice:** Implementing effective sustainment strategies may enhance the sustainment of chronic disease prevention programmes in both clinical and community settings.

**Policy:** To enhance long-term health outcomes, policy-makers should consider the sustainability of chronic disease prevention programmes early during initial implementation.

**Research:** Future research should focus on the development and empirical testing of strategies to address known barriers and facilitate the sustainment of health behaviour interventions, supporting positive long-term health outcomes.

## Introduction

Preventable chronic diseases, such as heart disease, diabetes, and respiratory disease, account for a significant proportion of morbidity and mortality globally [1]. There are several key behavioural risk factors associated with an increased risk of developing such chronic diseases including physical inactivity, poor diet, alcohol use, and tobacco use [2, 3]. A considerable proportion (2.78%–9.24%) of the total disease burden globally is attributed to these behavioural factors [4]. The World Health Organization recommends that evidence-based interventions (EBIs) be implemented routinely and equitably in clinical (e.g., hospitals, general practitioner surgeries, dental practices, community health centres) and community settings (e.g., schools, early childcare services, sporting clubs/organizations, and community centres) to reduce the prevalence of these risk factors. These settings are important as they provide centralized access to large proportions of the population [2] and often have access to existing infrastructure to support and enhance EBI delivery [5]. As such, governments internationally have made significant investments in the development and implementation of EBIs to address behavioural risk factors for chronic disease within these settings [6–8].

The sustainment of such EBIs is essential to ensure that population health benefits are achieved, the allocation of significant public health resources toward intervention delivery is protected, and community trust is not lost [9]. Sustainment is defined as ‘the extent the innovation is in place or being delivered long-term’ [10]. However, evidence suggests that after initial implementation support is withdrawn, ‘initiative decay’ is common [7]. For example, a systematic review by Stirman *et al.* [11], focused on the sustainment of public health and clinical interventions found that of 125 included studies, the majority of EBIs were only partially sustained following full initial implementation. Furthermore, systematic review evidence of the sustainment of school-based public health EBIs found that, of 18 included interventions, no intervention continued to be delivered in its entirety after the withdrawal of initial implementation support [12].

The sustainment of EBIs is an increasing concern for chronic disease prevention. Systematic reviews have identified several factors that impact the sustainment of EBIs in clinical and community settings including the availability of equipment, resources and facilities, continued executive or leadership support, and staff turnover [11, 13–15]. Strategies that address these determinants may be required to ensure that EBIs are sustained long-term and benefits are realized on a population level. While there is a growing body of evidence regarding the effectiveness of strategies that support initial implementation [6–8], only one review has collated strategies designed to explicitly support sustainment [16]. Of the 26 included studies, only six studies descriptively reported the use of strategies (nine strategies in total) designed to support sustainment [17–22]. The most reported strategies were the provision of funding and/or contracting for EBIs, and the continued use and maintenance of workforce skills through continued training, booster training sessions, supervision, and feedback [16]. However, this review did not synthesize any data relating to the effect or impact of sustainment strategies. Furthermore, there is a current gap in the generalizability of the evidence, as this review only focused on community settings.

To address these gaps, our primary aim was to determine the effect of strategies aiming to sustain the implementation of chronic disease prevention interventions (i.e., policies, practices, and programmes) in both clinical and community settings targeting key behavioural risk factors for chronic disease development (i.e., physical inactivity, poor diet, alcohol use, and tobacco use). Furthermore, when selecting sustainment strategies policy-makers and practitioners also need to know (1) whether sustainment strategies are also effective in sustaining the relevant health outcomes; (2) the cost to deliver sustainment strategies; and (3) if there are any unintended negative impacts from use of the strategies. However, policy-makers and practitioners are bereft of such information due to a scant evidence base. As such, our secondary aims were to (1) examine the effect of strategies designed to support sustainment on relevant health outcomes (including physical activity, healthy eating, obesity prevention, tobacco cessation, or alcohol use); (2) describe the cost implications of strategies designed to support sustainment; and (3) identify if there are any unintended or adverse effects of strategies designed to support sustainment on end-users.

## Materials and Methods

This systematic review was registered with PROSPERO and was undertaken according to the Cochrane Handbook for Systematic Reviews of Interventions [23], and is reported in accordance with the Preferred Reporting Items for Systematic review and Meta-Analysis checklist (PRISMA) [24], and Synthesis Without Meta-Analysis (SWiM) guidelines [25].

### Eligibility criteria

#### Study selection

We included any randomized controlled trial (RCT) which compared a strategy to sustain the implementation of a chronic disease prevention EBI in a clinical or community setting to no strategy or usual care (usual care may include some sustainment strategies provided that this constitutes as regular usual practice). Eligible study designs included RCT (with a parallel control group), cluster RCT (C-RCT; with a parallel control group and at least two clusters randomized to each group), stepped-wedge trial, or crossover design (only data prior to crossover will be used in the analysis). As there are variable definitions of sustainment within the literature and the length of time recommended to capture sustainment, there was no restriction on the length of the study follow-up period. There was also no restriction on language. Studies published in languages other than English were transcribed using an online transcription tool prior to their screening.

#### Types of participants

Eligible studies were those conducted within clinical settings (e.g., hospitals, general practitioner surgeries, community health centres) or community settings, for example, educational settings (i.e., primary and secondary schools, colleges, and universities), childcare services (i.e., long day care, family day care, preschools, and nurseries), elite or non-elite sports organizations and clubs (i.e., professional and amateur sports clubs, sporting governing bodies), and community centres (i.e., youth centres, community outreach centres). Study participants, therefore, included any person within these settings who may influence or have the responsibility for sustaining the implementation of the EBI. For example, in clinical settings, this could include clinicians, managers, allied health ,or other health service staff, whereas in community settings, it could include educational directors, principals, teachers, service managers, cooks/catering staff, coaches, and youth leaders.

#### Types of interventions

Studies must have compared a strategy or group of strategies following a successful period of implementation, to sustain the implementation of a chronic disease prevention intervention (i.e., tobacco cessation, healthy eating, physical activity, alcohol) to no sustainment strategy or usual care (this may include a ‘lower dose’ active sustainment strategy) to be eligible. We relied on the authors’ definition of whether or not implementation was successful, but programmes must have at minimum completed a period of initial implementation. Given the lack of standardised terminology on sustainment strategies, we also relied on definitions provided by authors regarding how they classified strategies. Examples of sustainment strategies include conducting ongoing training, distributing educational materials, and identifying and preparing champions.

##### Types of outcome measures—Primary outcome measures

studies that reported any objective or subjective measure of the ongoing delivery of physical activity, dietary, alcohol, or tobacco cessation interventions policy, practice, or programme within any of the eligible clinical or community settings were included. Sustained delivery of the EBI (e.g., proportion of schools continuing to deliver a healthy eating programme) must have been delivered by usual staff and not delivered by research personnel. Individual outcomes (e.g., sustained effects of patient participation in a programme, or individual’s maintenance of changes in health behaviour) were not considered sustainment outcomes. Outcome data may have been collected through surveys or questionnaires of staff (e.g., of their delivery of an EBI) or programme recipients (e.g., students or patients reported receipt of an EBI), audits of medical records, direct observation, or via routinely collected monitoring data.

#### Secondary outcome measures

Data on secondary outcomes were only extracted for those studies that first met the eligibility criteria for the primary review outcomes. Secondary outcomes included (1) health maintenance outcomes where an EBI is used to target modifiable behavioural risk factors related to chronic disease, that is, any objective or subjective measure of diet (e.g., fruit/vegetable intake), physical activity (e.g., minutes of physical activity during the school day), sedentary behaviour (e.g., daily minutes of sedentary time), weight status (e.g., body mass index), alcohol consumption (e.g., number of standard drinks consumed on a typical drinking day), and tobacco cessation (e.g., weekly number of cigarettes smoked). A hierarchy was used to prioritize multiple measures of the same health outcome (e.g., objective measures prioritized over subjective). (2) Cost outcomes relating to implementation costs, cost-effectiveness, or budget impact of strategies. (3) Any reported adverse effects of strategies. For example, negative impact on health outcomes (e.g., an increase in injury rates following physical activity initiatives), disruption to service operation or staff attitudes (e.g., negative impact on staff motivation or cohesion), or negative consequences to other key programmes or practices (e.g., lack of funding for other vital programs due to reallocation of funding). (4) Health equity outcomes, including any reported disparities in the reach or penetration of interventions among different population groups or settings over time, as well as adaptations made during the sustainment phase to promote health equity and address social determinants of health.

### Search strategy

A comprehensive search was developed in collaboration with an academic librarian (D.B.) using previously published search terms developed by co-authors (Supplementary File 1) [6–8]. Electronic database searches were conducted by D.B. from the earliest record to November 2022 in the Cochrane Central Register of Controlled trials (CENTRAL) via Cochrane Library; MEDLINE; PsycINFO and Embase via OVID; CINAHL via EBSCO; and SCOPUS via SCOPUS; Education Research Complete (November 2022) via EBSCO. There was no restriction on the language of publication. To identify additional relevant articles, the reference lists of all included studies and relevant systematic reviews were screened.

### Data collection and analysis

#### Study selection

**Figure 1:**
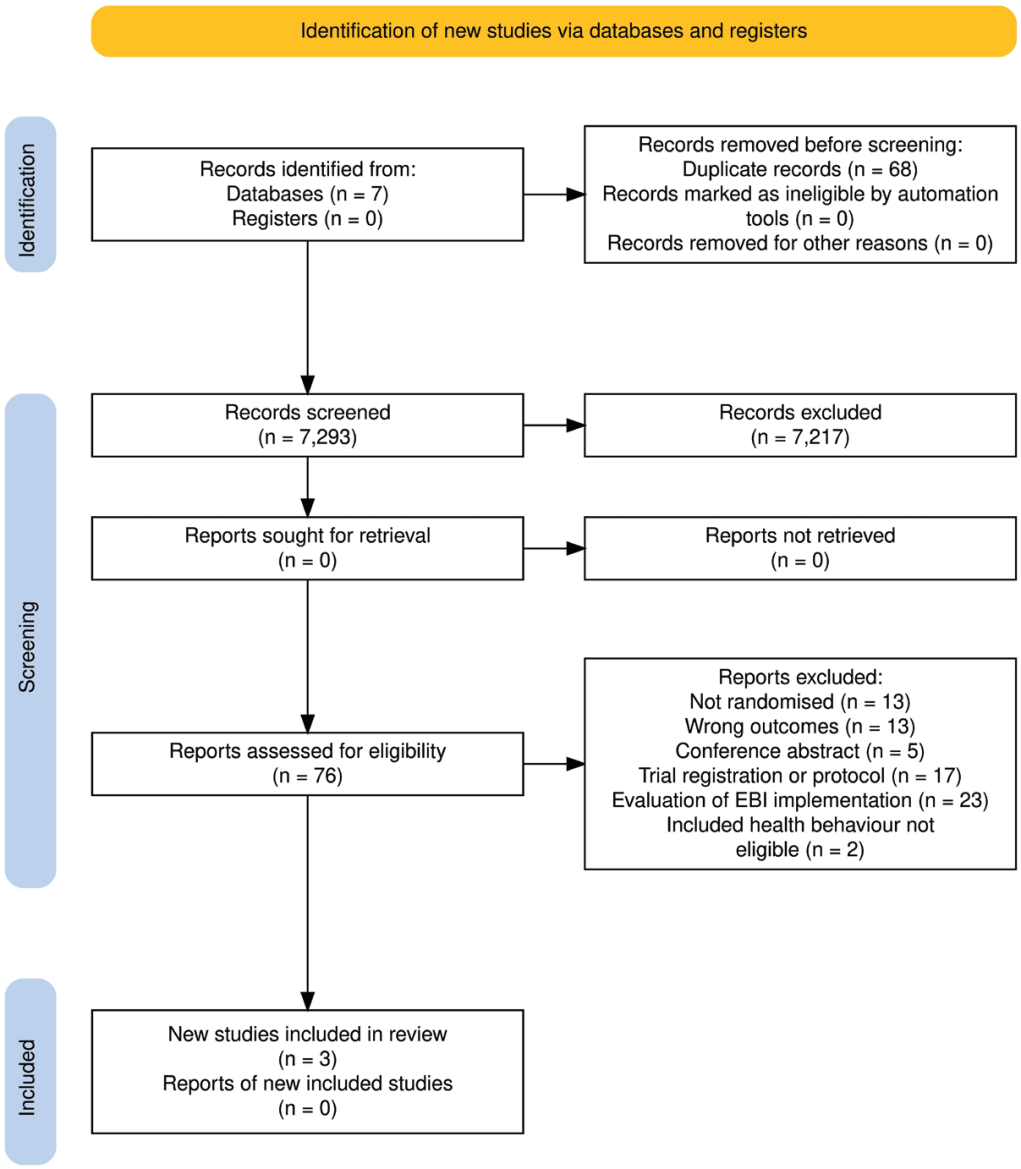
Preferred Reporting Items for Systematic Reviews and Meta-Analyses (PRISMA) study flow diagram

Title and abstract screening was conducted independently by pairs of review authors. Full texts of all potentially relevant studies were then obtained and assessed for eligibility according to the inclusion criteria, independently by pairs of review authors. Conflicts were resolved by consensus, and a third reviewer was consulted in any instances where consensus could not be reached. Figure 1 provides reasons for the exclusion of full text.

#### Data extraction and management

Two review authors (unblinded to author and journal information) independently extracted information from the included studies. Information extracted from the included studies was recorded in a data extraction form using Research Electronic Data Capture (REDCap) software [26, 27]. The data extraction form was developed based on the recommendations of the Cochrane Public Health Group Guide for Developing a Cochrane Protocol [23]. Data extraction discrepancies between review authors were resolved by consensus or by a third review author, if required. Information extracted from each of the studies included **‘**study eligibility’, ‘study design’, ‘date of publication’, ‘country the study was undertaken’, ‘description of the EBI’ including the health behaviour it was addressing, ‘what the intervention entailed and the setting it was delivered in’, ‘the duration of initial implementation support and length of time since withdrawn’ (if noted), ‘demographic/socioeconomic characteristics of the participants’, ‘the number of experimental conditions’, ‘overall study duration’, ‘time points measured’, ‘characteristics of the sustainment strategies’, that is, strategy description and duration for which the sustainment strategy was in place, theoretical underpinning, process evaluation measures of strategies (e.g., acceptability and appropriateness), primary and secondary outcomes of interest, and ‘information related to risk of bias assessment’.

#### Coding of strategies

Once extracted, strategies were independently double coded according to the sustainment-explicit Expert Recommendations for Implementing Change (ERIC) glossary [28]. The sustainment-explicit ERIC is a taxonomy that describes and organizes implementation and sustainment strategies. Any conflicts were resolved via consensus.

### Assessment of risk of bias in included studies

**Figure 2:**
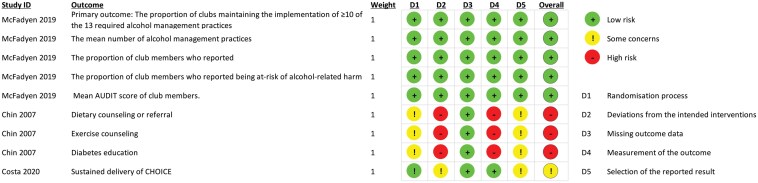
Risk-of-bias summary. D = Domain, AUDIT = Alcohol Use Disorder Identification Test

Two review authors assessed risk of bias independently for each review outcome using Version 2 of the Cochrane risk-of-bias tool for randomized trials (RoB 2). The RoB 2 Excel tool was used to implement RoB 2 (signalling questions were used for the following domains: bias arising from the randomization process, bias due to deviations from intended interventions, bias due to missing outcome data, bias in measurement of the outcome, bias in the selection of the reported result, and overall bias). The response options to the signalling questions were ‘yes’, ‘probably yes’, ‘probably no’, ‘no’, and ‘no information’. Once the signalling questions were answered, an RoB judgement and one of three levels (low RoB, some concerns, or high RoB) were assigned to each domain. An overall RoB was assigned to each study outcome, considering all the above domains. Overall RoB for study outcomes was assessed against set criteria and judged as per RoB 2 as: ‘low RoB’ *(the trial is judged to be at low RoB for all domains*), ‘some concerns’ (*the trial is judged to raise some concerns in at least one domain, but not be at high RoB for any domain*), and ‘high RoB’ (*the trial is judged to be at high risk of bias in at least on domain OR the trial is judged to have some concerns for multiple domains in a way that substantially lowers confidence in the result*) [23]. To determine if there was any reporting bias, we compared published reports to trial protocols and trial registries where available. Figure 2 outlines any potential for reporting bias and the RoB of the included studies.

### Data synthesis

If meta-analysis was undertaken, statistical heterogeneity was planned to be assessed by reviewing the distribution of studies using forest plots and assessing the *I*^2^ statistic. However, due to the low number of included studies, which makes it unlikely to provide accurate estimates of the between-study variation for random effects, meta-analysis, meta-analysis, and *I*^*2*^ testing were not conducted [29].

We used vote counting based on the direction of effect, following Cochrane guidelines [23]. This method allows us to summarize the overall trend of results from each study, even when the statistical significance of individual outcomes is not consistent. It’s important to note that vote counting summarizes the overall direction of the results and does not rely on the authors’ subjective opinion. Vote counting also does not consider the statistical significance or magnitude of individual outcomes. This means a study could show a ‘positive direction of effect’ even if its statistical outcomes were not significant. Vote counting based on statistical significance has been deemed unacceptable and has serious limitations [23]. Vote counting helps provide a general sense of the trends in the data but should be interpreted with caution, especially when the strength of evidence varies between studies. In our review, an upward-pointing arrow indicates a positive direction of effect (i.e., the intervention helped), and a downward-pointing arrow indicates a negative direction of effect (i.e., the intervention didn’t help). These are shown in Table 1.

**Table 1:** Strategies employed to sustain the delivery of EBIs

Study	Sustainability explicit ERIC strategy	Details of strategy	Mode of strategy delivery	Target of the strategy	Frequency of strategy delivery
Chin *et al.* [30]	Conduct ongoing training	Learning sessions that covered quality improvement topics, clinical subjects, and planning time.	Face-to-face	Health centre representatives (i.e., champions)	4 × 1.5-day learning sessions
	Use train-the-trainer strategies Identify and prepare champions	One member of staff diabetes from each health centre attended a week-long train-the-trainer course and then trained the rest of the staff in a sequence of learning sessions conducted over 4 months.	Face-to-face	Health centre representatives (champions) and staff	One week-long course followed by four 2-hour or eight 1-hour learning sessions conducted over 4 months
	Develop educational materials Distribute educational materials Involve patients/consumers and family members	14-Minute multilingual videos were created and shared with each centre. Videos were designed to empower patients to play an active role in their care.	Video	Patients	No information
	Organize clinician implementation team meetings	Sixteen 1-hour long conference calls were conducted with health centre representatives to discuss any issues, receive input, and feedback for the intervention, and to update health centres on the project.	Video call	Health centre representatives (champions)	Sixteen 1-hour conference calls
McFadyen *et al.* [31]	Identify and prepare champions	Each club assigned a champion who would be responsible for engaging with the web-based intervention and completing the online assessment and action plan.	Online (web-based programme)	Club champions	Champions had continuous access to the portal for 3 years
	Involve executive boards	Clubs were encouraged to review the alcohol management policy yearly with all executive members. Club executive members were automatically emailed results from their clubs’ online annual assessment along with the action plan.	Online (web-based programme)	Executive club members	Once annually during a yearly executive club meeting for 3 years
	Develop and organize quality monitoring systems Distribute educational materials Promote adaptability Conduct local needs assessment	Each club had access to their own online portal. Results from the online action plan automatically generated a tailored action plan for each club. The action plan included links to appropriate strategies and recourses for required actions. Clubs were able to adjust suggested action completion dates and were able to track their progress by updating the status of action completion.	Online (web-based programme)	Club champions	Assessment and action plan completed annually for 3 years. Club champions had access to the web-based portal continuously for 3 years.
	Provide audit and feedback	Clubs received tailored feedback based on their completion of the annual online assessment and completion of agreed actions. Feedback was provided via web-based programme and email.	Online (web-based programme)	Club champions	On completion of annual assessment and action tasks over 3 years
	Provide ongoing consultation	Help options were available via email and phone for user problems.	Telephone and email	Club champions	Continuously available for 3 years
	Develop educational materials	The web-based programme provided printable instruction manuals, sample policies, and planning templates.	Online (web-based program)	Club champions	Continuously available for 3 years
	Remind clinicians	Email reminder prompts were sent to prompt completion of the annual assessment and agreed actions.	Online (web-based programme)	Club champions	Reminders sent when the annual assessment was due for 3 years
	Involve patients/consumers and family members	Automatic notification and praise were sent to the club champion and club executives via email on completion of the assessment and action plan.	Online (web-based programme)	Club champions and executives	On completion of annual assessment and action plan for 3 years
Acosta *et al*. [32]	Develop educational materials Distribute educational materials Conduct local needs assessment Develop and implement tools for quality monitoring Remind clinicians	‘Getting to outcomes’ (GTO) Intervention manual was given to each site, which contained information, guidance, and tools for conducting needs assessments, goal setting, planning, process evaluations, assessments, and reminder prompts.	Physical manual document	Health centre staff	Given to sites at the start of the intervention
	Conduct ongoing training	Ongoing face-to-face training was conducted to guide staff in engaging with and completing the GTO steps	Face-to-face	Health centre staff	No information
	Provide local technical assistance	Ongoing, onsite, and proactive technical assistance was available to sites.	Face-to-face	Health centre staff	No information

To avoid over-representing studies that reported multiple results for the same outcome, we made sure each study only contributed one result per outcome. If a study reported more than one result for a specific outcome, we looked at the majority of results. For example, if more than 50% of the results from a single study suggested a positive effect for an outcome, we recorded that study as having a positive effect.

## Results

### Study selection

Following de-duplication, 7,293 records were included for title and abstract screening, of which 7217 were excluded. Of the remaining 76 articles, 73 were excluded as they did not meet our eligibility criteria; that is, non-randomized trials (*n* = 13), reported wrong outcomes (*n* = 13), trial registrations (*n* = 3), conference abstracts (*n* = 5), protocol publications (*n* = 14), evaluations of EBI implementation, not sustainability (*n* = 23), and included health behaviour, not eligible (*n* = 2). This left three included studies meeting our eligibility criteria [30–32]. Details are provided in Fig. 1.

### Study characteristics

A description of the included studies is shown in Table 2.

**Table 2:** Characteristics of the included studies

Study ID	Study design and sample	Country and setting	Year	EBI	Sustainment Intervention AND Control	Duration of initial implementation AND sustainment intervention	Final follow-up time point measured AND method of data collection	Primary outcomes of the review	Secondary outcomes of the review	Findings	Summary
Chin 2007 [30]	RCT Sample size: 34 health centres 17 intervention 17 control	USA community health centres	2007	Dietary and exercise counselling and diabetes education	Multi-component sustainment intervention Usual care (clinician implementation team meetings, and audit and feedback)	Initial implementation: 24 months Sustainment: 24 months	24 months Chart review instrument to review clinical records	% of patients who received: 1. Dietary counselling 2. Exercise counselling 3. Diabetes education	N/A	1. PO1: 0.24 (OR) [0.08, 0.68] 2. PO2: 0.34 (OR) [0.15, 0.75] 3. PO3: 0.16 (OR) [0.06, 0.44]	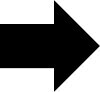
McFadyen 2019 [31]	RCT Sample size: 188 community sport clubs 92 intervention, 96 control	Australia Community football clubs	2019	Responsible alcohol management practices	Web-based multi-component sustainment intervention Usual care (one phone contact and reactive support provided on an as-needed basis)	Initial implementation: minimum of 12 months Sustainment: three consecutive football seasons over 29 months	25–27 months after baseline intervention implementation Field observations and Computer-Assisted Telephone Interviews	1. Proportion of clubs maintaining implementation of ≥10 of 13 alcohol management practices 2. Mean number of alcohol management practices implemented at follow-up	1. Proportion of club members who reported drinking alcohol at risky levels at sporting clubs 2. Proportion of club members who reported being at-risk of alcohol-related harm 3. Mean AUDIT score of club members	1. PO1: 0.53 (OR) [0.04, 7.2] *P* = 0.63 2. PO2: 0.1 (Mean difference) [−0.23, 0.42] *P* = 0.55 3. SO1: 0.71 (OR) [0.45, 1.10] *P* = 0.13 4. SO2: 1.12 (OR) [0.75, 1.67] *P* = 0.57 5. SO3: −0.04 (Mean difference) [−0.75, 0.67] *P* = 0.91	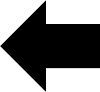 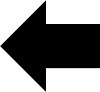
Acosta 2020 [32]	RCT Sample size: 29 afterschool programme centres 14 intervention, 15 control	USA afterschool programme centres	2020	Afterschool alcohol, cigarette and substance use prevention programme	Getting to outcomes implementation strategy	24 months	24 months Field observations	1. Staff adherence to delivering CHOICE (EBI)	N/A	1. PO1: 46% of intervention sites continued to deliver CHOICE, compared to 0% of control sites Fishers exact test *P* = 0.015^*^	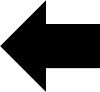

*Note*: Arrows indicate direction of effect (positive or negative effect on sustainment) and does not take into account statistical significance. Direction of effect was calculated based on available data reported in each paper. CHOICE = Name of the afterschool substance abuse; EBIPO = primary outcome; SO = secondary outcome.

^*^Statistical significance.

#### Types of studies

Of the three included studies, two were conducted in the USA [30, 32]., and one was conducted in Australia [31]. All three studies were RCTs [30–32]. Studies were conducted between 1998 and 2020.

#### Participants

One study was conducted in clinical settings (i.e., in 99 health centres in the USA) [30], and targeted health staff. Two of the studies were conducted in community settings, one in 188 non-elite level, Australian community football clubs in Australia (including Australian Football League, Rugby League, Rugby Union, or Soccer) targeting club members/affiliates [31], and the other in 29 afterschool sites in the US targeting club staff [32].

#### Types of interventions

The EBI of interest in each study targeted different health risk behaviours, with one study focused on providing dietary and exercise counselling, and diabetes education [30], the second focused on decreasing risky alcohol consumption, and the third focused on reducing targeted substance use (i.e., alcohol, e-cigarette, and other drug use) [32]. The length of delivery time for the employed sustainment strategies in the three studies ranged from 24 [30, 32] to 29 months [31]. All three studies employed multi-component sustainment strategies, with a total of 25 strategies used across the three studies (11 [31]; 7 [30]; 7 [32]). Two strategies (i.e., ‘develop educational materials’ and ‘distribute educational materials’) were employed in all three studies. Each study used a theoretical framework or model to develop strategies designed to support sustainment. These were (1) Sustainability Framework and the Persuasive Design framework [31], (2) Model for Improvement and the Chronic Care Model [30], and (3) The Consolidated Framework for Implementation Research [32].

### Risk of bias

The level of RoB for each study outcome is presented in Fig. 2. Overall RoB assessment for the primary outcome EBI sustainment deemed one study to be low RoB [31], one study was deemed to be high RoB [30], and the other was deemed to have some concerns [32]. All of the included studies were found to be at low RoB for ‘missing outcome data’ One study was found to be at high RoB for ‘deviations from the intended interventions’ due to no information being provided about participant or staff awareness of their assignment to intervention during the trial [30]. Furthermore, no information was provided regarding appropriate analysis to estimate the effect of assignment to intervention. In the same study, ‘measurement of the outcome’ was also high as there was no information provided on outcome assessor awareness of the intervention received by study participants [30]. One study was found to have some concerns for ‘deviations from the intended interventions’ due to no information being provided, and ‘selection of the reported result’ due to no availability of an analysis plan [32].

### Primary outcomes

#### Effect of strategies to support sustainment of EBIs

The effect of strategies designed to support sustainment varied across the three included studies. Nine outcomes from the three included studies (two [31], three [30], and one [32] respectively) contributed to the vote counting results. In the two studies that included multiple primary outcome measures, we combined the direction of effect for each outcome based on the direction in which most results for that outcome were found. For both studies, the majority of results were found to be positive (i.e., favouring the sustainment strategy) (three out of three results for the study by Chin *et al.* [30], and two out of two results for the study by McFadyen *et al.* [31],). Based on the vote-counting method, there was evidence that strategies designed to support sustainment positively impacted the sustainment of EBIs with two of three studies favouring the intervention [31, 32]. One study showed a negative effect on sustainment relative to the comparison group [30].

Chin *et al.* [30], aimed to sustain diabetes care in US community healthcare centres. Thirty-four healthcare centres were randomized into a standard -ntensity arm and a high-intensity arm. The high-intensity arm employed the following sustainment strategies: conduct ongoing training, use train-the-trainer strategies, identify and prepare champions, develop educational materials, distribute educational materials, involve patients/consumers and family members, and organize clinician implementation team meetings. The standard-intensity arm received organize clinician implementation team meetings and provide audit and feedback. Prior to randomization, all centres participated in the standard-intensity arm for a period of 2 years, which then provided baseline data for the sustainment intervention. The study reported on the sustainment of three primary outcomes measured via chart review of 80 randomly selected diabetic patients, 2 years after randomization. Follow-up data revealed a negative effect on sustainment of the following outcomes (percentage of patients who received): (1) dietary counselling or referral (odds ratio = 0.24 [95% confidence interval {CI}: 0.08, 0.68]); (2) exercise counselling: (odds ratio = 0.34 [95% CI: 0.15, 0.75]); and (3) diabetes education: (odds ratio = 0.16 [95% CI: 0.06, 0.44]). Based on vote counting, this study showed a negative effect on sustainment in the high-intensity intervention group relative to the standard-intensity control group.

McFadyen *et al.* [31]. aimed to sustain the implementation of responsible alcohol management practices in Australian community sports clubs [30]. This study compared two active strategies (intervention vs. usual support) to support sustainment. All clubs had previously participated in the Good Sports Program, and intervention clubs were supported to sustain the implementation of the alcohol management practices previously targeted by the club’s involvement in the Good Sports Program. The intervention employed the following strategies: identify and prepare champions, executive support targeting interactive intervention, tailored feedback, involve executive boards, develop and organize quality monitoring systems, distribute educational materials, promote adaptability, conduct local needs assessment, provide audit and feedback, provide ongoing consultation, develop educational materials, remind clinicians (club champions), use mass media, and involve patients/consumers and family members. Control clubs received the usual support provided to clubs accredited as Level 3 clubs with the Australian Drug Foundation’s Good Sports program which consisted of one phone contact and reactive support provided on an as-needed basis. Outcome data were collected via gold-standard field observations for implementation outcomes and Computer Assisted Telephone Interviews for health outcomes. At follow-up (25–27 months after baseline implementation of the sustainment intervention), there were no significant differences in sustainment of (1) the proportion of intervention (online support programme) clubs maintaining the implementation of ≥10 of the 13 required alcohol management practices (odds ratio = 0.53, 95% CI: 0.04, 7.2; *P* = 0.63), and (2) the mean number of alcohol management practices implemented at follow-up (mean difference = 0.1 [95% CI: −0.23, 0.42; *P* = 0.55]) compared to the comparison group. It is important to note that both the control and intervention groups were effective in maintaining the delivery of the EBI. However, based on vote counting, the intervention showed a positive effect on sustainment compared to control.

Finally, Acosta *et al*. [32]. aimed to sustain the delivery of an afterschool programme in the USA for preventing alcohol, cigarette, and substance use (known as CHOICE). The intervention group received support from a group of strategies called ‘Getting to Outcomes’ (GTO) and was compared to a control group (without GTO assistance). The sustainment strategies were integrated into the initial implementation of CHOICE, with GTO guiding both early delivery and long-term continuation planning. During the initial implementation, GTO steps 1–6 were covered, including needs assessment, goal setting, program planning, and assessing organizational capacity. To ensure sustainment, GTO steps 7–10 were implemented later, focusing on evaluation, quality improvement, and sustainability. The intervention employed the following strategies: develop educational materials, distribute educational materials, conduct local needs assessment, develop and implement tools for quality monitoring, remind clinicians, conduct ongoing training, and provide technical assistance. Follow-up (24 months after baseline) data revealed that 46% of centres sustained programme delivery (*P* = 0.015), compared to control sites, where none of the 12 sites continued their delivery of CHOICE. Based on vote counting, this study showed a positive effect on sustainment in the intervention group compared to the control group.

### Secondary outcomes

#### Health outcomes, cost outcomes, and any reported adverse effects of strategies

Only one study included data related to health maintenance outcomes. McFadyen *et al.* [31], investigated the impact of sustaining the implementation of responsible alcohol practices in Australian sporting clubs on three health-related outcomes. These outcomes were (1) the proportion of club members who reported drinking alcohol at risky levels at sporting clubs (odds ratio = 0.71, 95% CI: 0.45, 1.10, *P* = 0.13); (2) the proportion of club members who reported being at-risk of alcohol-related harm (odds ratio = 1.12, 95% CI: 0.75, 1.44, *P* = 0.57); and (3) mean AUDIT score of club members (mean difference = −0.04, 95% CI: −0.75, 0.67, *P* = 0.91). Based on vote counting, this study showed a positive direction of effect on health maintenance outcomes relating to alcohol consumption compared to the control group. The secondary outcome selected for this study was ‘the proportion of club members who reported drinking alcohol at risky levels at sporting clubs’.

No study included cost-related outcomes, nor did any study report on any adverse or unintended effects of strategies. None of the studies addressed health equity considerations in their sustainment strategies.

## Discussion

Despite overwhelming evidence demonstrating the need to sustain chronic disease prevention interventions, this review identified only three controlled trials that sought to test the effectiveness of strategies designed to support the sustainment of EBIs in clinical and community settings. The predominant focus in public health research on developing and testing the efficacy of interventions, often at the expense of understanding how to effectively implement and sustain these interventions, has resulted in a scarcity of sustainment-focused research. Based on vote counting for the primary outcomes, two of the included studies favoured the sustainment intervention [31, 32], and one study favoured the control [30]. Only one study reported on a secondary outcome (maintenance of health behaviour), which found a favourable result, favouring the intervention [31]. This was also the only study found to have an overall low RoB for all primary and secondary outcomes [31]. This review highlights the urgent need for more rigorous research on effective strategies to sustain implementation. This scarcity of research not only hinders research efforts to deepen our understanding of sustainability but also impacts policy-makers and practitioners’ ability to successfully translate evidence into practice. By calling attention to this issue, we hope to better advocate for the integration of sustainment strategies into intervention design, ensuring that EBIs can be sustained in both clinical and community settings.

Based on vote counting, we found evidence of a positive effect of sustainment strategies in two of the three included studies [31, 32]. The primary difference between the studies which showed a positive effect [31, 32] versus a negative effect [30] on sustainment may be due to Chin *et al*. [30], predominantly focusing on only one known barrier to sustainment (i.e., implementer skills/expertise) [33], whilst the other two studies [31, 32] targeted a broader range of barriers. Four out of the six included strategies used by Chin *et al.* [30], focused on implementer skills/expertise (i.e., conduct ongoing training, use train-the-trainer strategies, identify and prepare champions, etc.). While this domain is a significant barrier to sustaining EBIs [33–35], it is just one factor among many [33]. Theoretical frameworks [33] as well as findings of systematic reviews [11, 12, 34, 36] highlight the complexity of factors that influence the sustainment of EBIs. These include the characteristics of the innovation itself, the attributes of those implementing it, the specific delivery setting, and broader external contextual factors. Thus, the multi-strategy approach adopted by McFadyen *et al*. [31], and Acosta *et al.* [32] is likely to have addressed a wide range of sustainment determinants potentially explaining why their interventions showed some positive effects on sustainment outcomes compared to Chin *et al*. [30].

However, the results of the study by Chin *et al*. [30] should be viewed with caution. However, the high-intensity intervention was associated with less documentation of diabetes education and dietary and exercise counselling compared to the standard-intensity comparison group. There were observed increases in the sustainment of clinical outcomes (i.e., medication use) regarding diabetes management, compared to the standard-intensity comparison group. These outcomes were outside the scope of this review, but this suggests there may be trade-offs between increasing medication use and participating in diabetes education and dietary and exercise counselling. Additionally, the authors argue that behavioural outcomes are more prone to documentation variation than more clinical outcomes [30]. Furthermore, the standard-intensity group received clinician implementation team meetings, and audit and feedback, which may be more effective in this setting, possibly due to these strategies being less of a burden on staff compared to the strategies used in the high-intensity intervention group.

The most commonly reported strategies in the present review were ‘develop educational materials’, ‘distribute educational materials’, ‘identify and prepare champions’, and ‘conduct ongoing training’, which target the contextual factors, processes, and characteristics of the interventionists domains of the integrated sustainability framework [33]. This finding shares similarities with the previous review [16], which also found the most frequently reported strategies targeted these same domains. The most commonly reported strategies by Hailermariam *et al*. [16] were ‘maintenance of workforce skills through continued training’, ‘booster training sessions’, ‘supervision and feedback’, and ‘organizational leader stakeholder prioritizing and supporting continued use’. As such, strategies that focus on addressing these domains may be of particular importance and warrant additional consideration when designing strategies to support sustainment.

The review by Hailemariam *et al*. [16] included 26 studies, none of which were included in the current review. This is due to our strict eligibility criteria, focusing on health behaviours and including only studies with an RCT, stepped-wedge, and crossover designs. We employed this approach to align with our research questions, and for pragmatic reasons to ensure data were manageable. The eligible research designs that we chose are considered as the gold standard for assessing causal effects [37], so are most appropriate for addressing the research question. Furthermore, the health behaviours that we chose are the leading risk factors for the development of chronic disease [3]. Future reviews that broaden the search to include additional health behaviours and/or include a greater range of research designs may find an increased number of eligible studies to allow for meta-analysis.

The present review found no included strategies related to funding, despite the prevalence of these strategies in the review by Hailemariam *et al*. [21], and funding being a key barrier to sustainment [34]. The lack of funding strategies in the present review may be explained as the included studies purposely designed strategies to accommodate for a delivery context with limited funding, and so did not include strategies to target funding issues. This is evident as two of the included studies, conducted in low-resourced community settings, explicitly stated their intent to select low-cost strategies [30, 31]. As such, funding remains a considerable challenge for sustainment, and strategies which target this key barrier are likely to be important for EBI sustainment.

The public health value of sustaining EBIs is only realized when EBIs continue to deliver equitable positive health impacts and demonstrate cost-effectiveness over time [33, 34]. As McFadyen *et al*. [31] was the only study to report on the health impact of sustained delivery of their EBI, and none of the included studies reported on cost or adverse events; thus restricting our ability to report any potential impact of sustainment efforts, positive or negative. Policy-makers, practitioners, researchers, funders, and health leaders require this information to assess the sustained public health impact of these programmes in relation to the ongoing cost of their delivery, informing decisions of where to continue, replace, or cease EBI delivery altogether. Hybrid type 2 trials, which simultaneously assess the effectiveness of a strategy and its health impact [38], could be useful designs to employ to build the evidence base more rapidly. None of the included studies addressed health equity, highlighting a significant gap in the literature. Sustaining interventions is particularly important for systemically disadvantaged populations [39], and future research must focus on strategies that effectively support these communities. Advancing health equity requires sustainment strategies that reduce inequities, such as building trust, partnerships, capacity, and advocating for allyship and adaptations [40]. Furthermore, to facilitate replication and evidence synthesis of sustainment strategies, we recommend that standardized terminology be used, in line with the sustainment-explicit ERIC glossary [28], and that strategies are reported in combination with frameworks such as the Action, Actor, Context, Target, Time (AACTT) [41] and Proctor *et al*. [42] strategy reporting guidelines.

### Limitations

Despite our best efforts, there were also limitations identified through our review process. As we only included studies with controlled designs, there may be other non-controlled studies that were not detected that may have provided important learnings. Furthermore, we were unable to conduct a meta-analysis that would provide a more comprehensive understanding of the effect of sustainment strategies. Lastly, although we coded strategies according to the Sustainment-Explicit ERIC glossary [28], due to inconsistent reporting of strategies and lack of detail regarding included strategies, the coding may be subject to individual interpretation.

## Conclusion

Given the challenges of undertaking sustainability research, it is not surprising that so few studies were found. Our review highlights the importance of a concerted effort to advance the field of sustainability science. Future research, which employs robust designs and comprehensively describes the use and empirical testing of sustainment strategies will greatly contribute to the knowledge base in terms of understanding which strategies are more effective, is supporting EBI sustainment across clinical and community settings.

## Supplementary Material

ibae070_suppl_Supplementary_Files_1

ibae070_suppl_Supplementary_Checklist

## Data Availability

All data collected from individual studies are fully reported within the systematic review manuscript.

## Notes

Registration: This review was prospectively registered with PROSPERO (registration ID: CRD42022352333. August 2022)

## Welfare of Animals

This article does not contain any studies with animals performed by any of the authors.

## Transparency Statements

**Study Registration**

This review was prospectively registered with PROSPERO (registration ID: CRD42022352333, August 2022).

**Analytic Plan Pre-registration**

The analytic plan was registered prior to beginning data collection with PROPERSO (registration ID: CRD42022352333, August 2022).

**Analytic code availability**

There is no analytic code associated with this study.

**Materials availability**

All materials including the search strategy and PRISMA checklist are available by emailing the corresponding author.
