# Supplementary material for: A systematic review to determine the effect of strategies to sustain chronic disease prevention interventions in clinical and community settings
Source: Transl Behav Med. 2025 Jan 22;15(1):ibae070. doi: 10.1093/tbm/ibae070 (PMC11752859; doi:10.1093/tbm/ibae070)
Supplement: ibae070_suppl_Supplementary_Files_1 [file ibae070_suppl_supplementary_files_1.docx]

Sustainability Review

Searches conducted: 6^th^-7^th^ November 2022

| **Database** | **Vendor** | **Records identified** | **Records after de-duplication** |
| --- | --- | --- | --- |
| CENTRAL | Wiley | 1672 | 800 |
| CINAHL Complete | EBSCO | 1731 | 921 |
| Embase | OVID | 3165 | 1581 |
| Education Research Complete | EBSCO | 1042 | 781 |
| Medline | OVID | 1926 | 1919 |
| PsycINFO | OVID | 804 | 359 |
| Scopus | Scopus | 3103 | 998 |
| **TOTAL** |  | **13,443** | **7,361** |

Database(s): **Ovid MEDLINE(R) and Epub Ahead of Print, In-Process, In-Data-Review & Other Non-Indexed Citations and Daily**1946 to November 04, 2022
Search Strategy:

| **#** | **Searches** | **Results** |
| --- | --- | --- |
| 1 | exp obesity/ | 250906 |
| 2 | Weight Gain/ | 34662 |
| 3 | exp Weight Loss/ | 48399 |
| 4 | exp Exercise/ | 237542 |
| 5 | Motor Activity/ | 99822 |
| 6 | "Physical Education and Training"/ | 14115 |
| 7 | Physical Fitness/ | 29353 |
| 8 | exp Life Style/ | 108259 |
| 9 | exp Leisure Activities/ | 269443 |
| 10 | sport*.tw,kw. | 97539 |
| 11 | ((life style or life style) adj5 activ*).tw,kw. | 483 |
| 12 | exp Diet/ | 320164 |
| 13 | nutrition*.mp. | 449611 |
| 14 | (health* adj2 eat*).tw,kw. | 12074 |
| 15 | Child Nutrition Sciences/ | 1178 |
| 16 | Vegetables/ or vegetable*.tw,kw. | 77509 |
| 17 | Food Services/ | 6090 |
| 18 | menu*.tw,kw. | 5868 |
| 19 | Eating/ | 58381 |
| 20 | Food Habits/ | 91304 |
| 21 | (school adj2 (lunch* or meal*)).tw,kw. | 2058 |
| 22 | Menu Planning/ | 1449 |
| 23 | (nutrition* adj2 program*).mp. | 7458 |
| 24 | cafeteria*.tw,kw. | 2308 |
| 25 | exp Smoking/ | 159007 |
| 26 | exp "Tobacco Use Cessation"/ | 1408 |
| 27 | Nicotine / | 27715 |
| 28 | Tobacco/ or "Tobacco Use"/ | 37514 |
| 29 | ((ceas* or cess* or prevent* or stop* or quit* or abstin* or abstain* or reduc*) adj5 (smok* or tobacco or nicotine)).tw,kw. | 63578 |
| 30 | alcohol drinking/ or binge drinking/ | 75422 |
| 31 | alcohol*.tw,kw. | 383161 |
| 32 | Alcoholic Intoxication/ or Alcoholism/ | 89777 |
| 33 | (alcohol* or drink* or liquor* or beer* or wine* or spirit* or drunk* or intoxicat* or binge*).tw,kw. | 600265 |
| 34 | or/1-33 | 2315476 |
| 35 | controlled clinical trial/ or randomized controlled trial/ | 670135 |
| 36 | group*.tw. | 4330866 |
| 37 | trial.ti. | 272969 |
| 38 | randomi?ed.ab. | 693838 |
| 39 | clinical trials as topic.sh. | 200534 |
| 40 | placebo.ab. | 232905 |
| 41 | randomly.ab. | 394524 |
| 42 | stepped wedge.mp. | 1470 |
| 43 | or/35-42 | 5160102 |
| 44 | ((daycare or day care or school* or universit* or community health or church* or community outreach or youth cent* or hospital* or inpatient* or outpatient* or general practice or family practice or workplace or work place or primary care or club* or public health) adj3 (sustain* or implement* or routini* or durabil* or embed* or integrat* or normali* or institutionali* or maintain* or continu* or adher* or stabili*)).mp | 41119 |
| 45 | 34 and 43 and 44 | 1931 |
| 46 | exp animals/ not humans/ | 5060776 |
| 47 | 45 not 46 | 1926 |

Database(s): **Embase**1947 to present
Search Strategy:

| **#** | **Searches** | **Results** |
| --- | --- | --- |
| 1 | obesity/ | 515532 |
| 2 | body weight gain/ | 32309 |
| 3 | body weight loss/ | 72400 |
| 4 | exercise/ | 350373 |
| 5 | motor activity/ | 52502 |
| 6 | physical education/ | 15397 |
| 7 | fitness/ | 44288 |
| 8 | exp lifestyle/ | 154514 |
| 9 | leisure/ | 44991 |
| 10 | sport*.tw,kw. | 130092 |
| 11 | ((life style or life style) adj5 activ*).tw,kw. | 841 |
| 12 | exp diet/ | 427499 |
| 13 | nutrition*.mp. | 628762 |
| 14 | (health* adj2 eat*).tw,kw. | 15947 |
| 15 | nutritional science/ | 6487 |
| 16 | Vegetable/ or vegetable*.tw,kw. | 98468 |
| 17 | catering service/ | 22002 |
| 18 | menu*.tw,kw. | 7559 |
| 19 | eating/ | 43274 |
| 20 | feeding behavior/ | 97395 |
| 21 | (school adj2 (lunch* or meal*)).tw,kw. | 2614 |
| 22 | (nutrition* adj2 program*).mp. | 9502 |
| 23 | cafeteria*.tw,kw. | 2899 |
| 24 | exp smoking/ | 458429 |
| 25 | nicotine/ | 53863 |
| 26 | tobacco/ or "tobacco use"/ | 70658 |
| 27 | ((ceas* or cess* or prevent* or stop* or quit* or abstin* or abstain* or reduc*) adj5 (smok* or tobacco or nicotine)).tw,kw. | 83070 |
| 28 | drinking behavior/ | 56356 |
| 29 | binge drinking/ | 7040 |
| 30 | alcohol*.tw,kw. | 549245 |
| 31 | alcoholism/ | 143587 |
| 32 | (alcohol* or drink* or liquor* or beer* or wine* or spirit* or drunk* or intoxicat* or binge*).tw,kw. | 846806 |
| 33 | 1 or 2 or 3 or 4 or 5 or 6 or 7 or 8 or 9 or 10 or 11 or 12 or 13 or 14 or 15 or 16 or 17 or 18 or 19 or 20 or 21 or 22 or 23 or 24 or 25 or 26 or 27 or 28 or 29 or 30 or 31 or 32 | 3339482 |
| 34 | clinical trial/ or controlled clinical trial/ | 1136583 |
| 35 | randomized controlled trial/ | 737708 |
| 36 | group*.tw. | 6243056 |
| 37 | trial.ti. | 382140 |
| 38 | randomi?ed.ab. | 1005154 |
| 39 | placebo.ab. | 344380 |
| 40 | randomly.ab. | 525436 |
| 41 | stepped wedge.mp. | 1865 |
| 42 | 34 or 35 or 36 or 37 or 38 or 39 or 40 or 41 | 7664723 |
| 43 | ((daycare or day care or school* or universit* or community health or church* or community outreach or youth cent* or hospital* or inpatient* or outpatient* or general practice or family practice or workplace or work place or primary care or club* or public health) adj3 (sustain* or implement* or routini* or durabil* or embed* or integrat* or normali* or institutionali* or maintain* or continu* or adher* or stabili*)).mp. | 58915 |
| 44 | 33 and 42 and 43 | 3179 |
| 45 | exp animal/ not human/ | 5972233 |
| 46 | 44 not 45 | 3165 |

Database(s): **APA PsycInfo**1806 to October Week 4 2022
Search Strategy:

| **#** | **Searches** | **Results** |
| --- | --- | --- |
| 1 | obesity/ | 27717 |
| 2 | weight gain/ | 3461 |
| 3 | weight loss/ | 4398 |
| 4 | exp exercise/ | 30731 |
| 5 | Physical Education/ or Physical Activity/ | 29191 |
| 6 | Physical Fitness/ | 4757 |
| 7 | exp lifestyle/ | 13454 |
| 8 | Leisure Time/ | 6879 |
| 9 | ((life style or life style) adj5 activ*).tw,id. | 111 |
| 10 | exp diets/ | 19395 |
| 11 | sport*.tw,id. | 41487 |
| 12 | nutrition*.mp. | 37057 |
| 13 | (health* adj2 eat*).tw,id. | 5040 |
| 14 | Nutrition/ | 12516 |
| 15 | vegetable*.mp. | 6974 |
| 16 | Food Service*.mp. | 1186 |
| 17 | menu*.tw,id. | 2618 |
| 18 | Eating Behavior/ | 15902 |
| 19 | eating.mp. | 72691 |
| 20 | food habit*.mp. | 3874 |
| 21 | (school adj2 (lunch* or meal*)).tw,id. | 804 |
| 22 | cafeteria*.tw,id. | 836 |
| 23 | Smoking.mp. or Tobacco Smoking/ | 65449 |
| 24 | Tobacco Smoking/ | 35003 |
| 25 | Nicotine/ | 12367 |
| 26 | ((ceas* or cess* or prevent* or stop* or quit* or abstin* or abstain* or reduc*) adj5 (smok* or tobacco or nicotine)).tw,id. | 25943 |
| 27 | Drinking Behavior/ or Binge Drinking/ or Animal Drinking Behavior/ | 7403 |
| 28 | alcohol*.tw,id. | 143305 |
| 29 | alcoholism/ or alcohol intoxication/ | 33628 |
| 30 | (alcohol* or drink* or liquor* or beer* or wine* or spirit* or drunk* or intoxicat* or binge*).tw,id. | 230571 |
| 31 | 1 or 2 or 3 or 4 or 5 or 6 or 7 or 8 or 9 or 10 or 11 or 12 or 13 or 14 or 15 or 16 or 17 or 18 or 19 or 20 or 21 or 22 or 23 or 24 or 25 or 26 or 27 or 28 or 29 or 30 | 487268 |
| 32 | clinical trials/ or randomized controlled trials/ | 13024 |
| 33 | group*.tw. | 1001030 |
| 34 | trial.ti. | 36231 |
| 35 | randomi?ed.ab. | 93014 |
| 36 | placebo.ab. | 42315 |
| 37 | randomly.ab. | 80985 |
| 38 | stepped wedge.mp. | 169 |
| 39 | 32 or 33 or 34 or 35 or 36 or 37 or 38 | 1112157 |
| 40 | ((daycare or day care or school* or universit* or community health or church* or community outreach or youth cent* or hospital* or inpatient* or outpatient* or general practice or family practice or workplace or work place or primary care or club* or public health) adj3 (sustain* or implement* or routini* or durabil* or embed* or integrat* or normali* or institutionali* or maintain* or continu* or adher* or stabili*)).mp. | 25063 |
| **41** | **31 and 39 and 40** | **804** |

**CINAHL Complete**

| **#** | **Query** | **Results** |
| --- | --- | --- |
| S1 | (MH "Obesity+") | 113,662 |
| S2 | (MH "Weight Gain") | 13,607 |
| S3 | (MH "Weight Loss+") | 26,559 |
| S4 | (MH "Exercise+") | 129,304 |
| S5 | (MH "Motor Activity") | 12,561 |
| S6 | (MH "Physical Education and Training") | 4,016 |
| S7 | (MH "Physical Fitness") | 19,299 |
| S8 | (MH "Life Style+") | 267,603 |
| S9 | (MH "Leisure Activities+") | 79,492 |
| S10 | TI sport* OR AB sport* OR MW sport* | 86,127 |
| S11 | TI ( ((life style or life style) n5 activ*) ) OR AB ( ((life style or life style) n5 activ*) ) OR MW ( ((life style or life style) n5 activ*) ) | 172 |
| S12 | (MH "Diet+") | 139,724 |
| S13 | (MH "Nutrition") OR "nutrition*" | 185,817 |
| S14 | TI (health* n2 eat*) OR AB (health* n2 eat*) OR MW (health* n2 eat*) | 10,448 |
| S15 | (MH "Child Nutrition") | 7,985 |
| S16 | (MH "Vegetables") OR "vegetable*" | 28,348 |
| S17 | (MH "Food Services") | 7,572 |
| S18 | (MH "Menu Planning") OR "menu*" | 4,786 |
| S19 | (MH "Eating") OR (MH "Eating Behavior") OR (MH "Food Habits") | 41,268 |
| S20 | TI ( (school n2 (lunch* or meal*)) ) OR AB ( (school n2 (lunch* or meal*)) ) OR MW ( (school n2 (lunch* or meal*)) ) | 1,619 |
| S21 | TI (nutrition* n2 program*) OR AB (nutrition* n2 program*) OR MW (nutrition* n2 program*) | 5,065 |
| S22 | "cafeteria*" | 810 |
| S23 | (MH "Smoking+") | 78,654 |
| S24 | (MH "Nicotine") | 5,454 |
| S25 | (MH "Tobacco") | 9,040 |
| S26 | TI ( ((ceas* or cess* or prevent* or stop* or quit* or abstin* or abstain* or reduc*) n5 (smok* or tobacco or nicotine)) ) OR AB ( ((ceas* or cess* or prevent* or stop* or quit* or abstin* or abstain* or reduc*) n5 (smok* or tobacco or nicotine)) ) OR MW ( ((ceas* or cess* or prevent* or stop* or quit* or abstin* or abstain* or reduc*) n5 (smok* or tobacco or nicotine)) ) | 44,584 |
| S27 | (MH "Alcohol Drinking") OR (MH "Binge Drinking") | 35,053 |
| S28 | TI alcohol* OR AB alcohol* OR MW alcohol* | 119,890 |
| S29 | (MH "Alcoholism") | 18,489 |
| S30 | TI ( (alcohol* or drink* or liquor* or beer* or wine* or spirit* or drunk* or intoxicat* or binge*) ) OR AB ( (alcohol* or drink* or liquor* or beer* or wine* or spirit* or drunk* or intoxicat* or binge*) ) OR MW ( (alcohol* or drink* or liquor* or beer* or wine* or spirit* or drunk* or intoxicat* or binge*) ) | 188,954 |
| S31 | S1 OR S2 OR S3 OR S4 OR S5 OR S6 OR S7 OR S8 OR S9 OR S10 OR S11 OR S12 OR S13 OR S14 OR S15 OR S16 OR S17 OR S18 OR S19 OR S20 OR S21 OR S22 OR S23 OR S24 OR S25 OR S26 OR S27 OR S28 OR S29 OR S30 | 1,067,809 |
| S32 | (MH "Clinical Trials") OR (MH "Randomized Controlled Trials") | 307,031 |
| S33 | TI group* OR AB group* OR MW group* | 1,028,960 |
| S34 | TI trial | 172,057 |
| S35 | AB randomi?ed | 248,840 |
| S36 | AB placebo | 65,937 |
| S37 | AB randomly | 106,898 |
| S38 | "stepped wedge" | 965 |
| S39 | S32 OR S33 OR S34 OR S35 OR S36 OR S37 OR S38 | 1,351,652 |
| S40 | TI ( ((daycare or "day care" or school* or universit* or "community health" or church* or "community outreach" or "youth cent*" or hospital* or inpatient* or outpatient* or "general practice" or "family practice" or workplace or "work place" or "primary care" or club* or "public health") n3 (sustain* or implement* or routini* or durabil* or embed* or integrat* or normali* or institutionali* or maintain* or continu* or adher* or stabili*)) ) OR AB ( ((daycare or "day care" or school* or universit* or "community health" or church* or "community outreach" or "youth cent*" or hospital* or inpatient* or outpatient* or "general practice" or "family practice" or workplace or "work place" or "primary care" or club* or "public health") n3 (sustain* or implement* or routini* or durabil* or embed* or integrat* or normali* or institutionali* or maintain* or continu* or adher* or stabili*)) ) OR MW ( ((daycare or "day care" or school* or universit* or "community health" or church* or "community outreach" or "youth cent*" or hospital* or inpatient* or outpatient* or "general practice" or "family practice" or workplace or "work place" or "primary care" or club* or "public health") n3 (sustain* or implement* or routini* or durabil* or embed* or integrat* or normali* or institutionali* or maintain* or continu* or adher* or stabili*)) ) | 31,395 |
| **S41** | **S31 AND S39 AND S40 Humans** | **1,731** |

**CENTRAL**

**ID Search Hits**

#1 MeSH descriptor: [Obesity] explode all trees 16166

#2 MeSH descriptor: [Weight Gain] this term only 2762

#3 MeSH descriptor: [Weight Loss] explode all trees 7239

#4 MeSH descriptor: [Exercise] explode all trees 28878

#5 MeSH descriptor: [Motor Activity] this term only 3783

#6 MeSH descriptor: [Physical Education and Training] this term only 1650

#7 MeSH descriptor: [Physical Fitness] this term only 3075

#8 MeSH descriptor: [Life Style] explode all trees 6381

#9 MeSH descriptor: [Leisure Activities] explode all trees 20506

#10 sport*:ti,ab,kw 12023

#11 ((life style or life style) near/5 activ*):ti,ab,kw 12986

#12 MeSH descriptor: [Diet] explode all trees 20505

#13 nutrition* 78199

#14 (health* near/2 eat*):ti,ab,kw 3096

#15 MeSH descriptor: [Child Nutrition Sciences] this term only 143

#16 vegetable*:ti,ab,kw 7643

#17 MeSH descriptor: [Vegetables] this term only 1411

#18 MeSH descriptor: [Food Services] this term only 278

#19 menu*:ti,ab,kw 4438

#20 MeSH descriptor: [Eating] this term only 3028

#21 MeSH descriptor: [Feeding Behavior] this term only 3547

#22 (school near/2 (lunch* or meal*)):ti,ab,kw 431

#23 MeSH descriptor: [Menu Planning] this term only 45

#24 (nutrition* near/2 program*):ti,ab,kw 1499

#25 cafeteria*:ti,ab,kw 220

#26 MeSH descriptor: [Smoking] explode all trees 6548

#27 MeSH descriptor: [Tobacco Use Cessation] explode all trees 128

#28 MeSH descriptor: [Nicotine] this term only 2839

#29 MeSH descriptor: [Tobacco] this term only 212

#30 MeSH descriptor: [Tobacco Use] this term only 84

#31 ((ceas* or cess* or prevent* or stop* or quit* or abstin* or abstain* or reduc*) near/5 (smok* or tobacco or nicotine)):ti,ab,kw 16796

#32 MeSH descriptor: [Alcohol Drinking] this term only 4124

#33 MeSH descriptor: [Binge Drinking] this term only 187

#34 alcohol*:ti,ab,kw 35644

#35 MeSH descriptor: [Alcoholic Intoxication] this term only 687

#36 MeSH descriptor: [Alcoholism] this term only 3987

#37 (alcohol* or drink* or liquor* or beer* or wine* or spirit* or drunk* or intoxicat* or binge*):ti,ab,kw 54577

#38 {OR #1-#37} 223353

#39 ((daycare or "day care" or school* or universit* or "community health" or church* or "community outreach" or "youth cent*" or hospital* or inpatient* or outpatient* or "general practice" or "family practice" or workplace or "work place" or "primary care" or club* or "public health") near/3 (sustain* or implement* or routini* or durabil* or embed* or integrat* or normali* or institutionali* or maintain* or continu* or adher* or stabili*)) 8036

**#40 {AND #38, #39} 2065 (1672 TRIALS)**

**Education Research Complete**

AB ( (obesity or “weight gain” or “weight loss” or exercise or “motor activity” or “physical education” or “physical fitness” or “life style” or lifestyle or “leisure activit*” or sport* or diet or nutrition* or (health* and eat*) or vegetable* or “food service*” or menu* or eating or “food habit*” or “school lunch*” or “school meal*” or cafeteria* or smoking or tobacco or nicotine or alcohol* or drink* or liquor* or beer* or wine* or spirit* or drunk* or intoxicat* or binge*) ) AND AB ( Group* or trial or random* or placebo or “stepped wedge” ) AND AB ( ((daycare or “day care” or school* or universit* or “community health” or church* or “community outreach” or “youth cent*” or hospital* or inpatient* or outpatient* or “general practice” or “family practice” or workplace or “work place” or “primary care” or club* or “public health”) n3 (sustain* or implement* or routini* or durabil* or embed* or integrat* or normali* or institutionali* or maintain* or continu* or adher* or stabili*)) )

**Scopus**

ABS ( ( obesity OR "weight gain" OR "weight loss" OR exercise OR "motor activity" OR "physical education" OR "physical fitness" OR "life style" OR lifestyle OR "leisure activit*" OR sport* OR diet OR nutrition* OR ( health* AND eat* ) OR vegetable* OR "food service*" OR menu* OR eating OR "food habit*" OR "school lunch*" OR "school meal*" OR cafeteria* OR smoking OR tobacco OR nicotine OR alcohol* OR drink* OR liquor* OR beer* OR wine* OR spirit* OR drunk* OR intoxicat* OR binge* ) ) AND ABS ( group* OR trial OR random* OR placebo OR "stepped wedge" ) AND TITLE-ABS-KEY ( ( ( daycare OR "day care" OR school* OR universit* OR "community health" OR church* OR "community outreach" OR "youth cent*" OR hospital* OR inpatient* OR outpatient* OR "general practice" OR "family practice" OR workplace OR "work place" OR "primary care" OR club* OR "public health" ) W/3 ( sustain* OR implement* OR routini* OR durabil* OR embed* OR integrat* OR normali* OR institutionali* OR maintain* OR continu* OR adher* OR stabili* ) ) ) AND ( LIMIT-TO ( EXACTKEYWORD , "Human" ) OR LIMIT-TO ( EXACTKEYWORD , "Humans" ) )
